# Longitudinal program evaluation of an inter-institutional mentorship network for pediatric rheumatology using a quality improvement framework

**DOI:** 10.21203/rs.3.rs-3717708/v1

**Published:** 2023-12-12

**Authors:** Kristen Hayward, Alexi Grom, Eyal Muscal, Peter A. Nigrovic, Kelly A. Rouster-Stevens, Kaveh Ardalan, Linda Hiraki, L. Nandini Moorthy

**Affiliations:** Seattle Children’s Hospital and University of Washington School of Medicine; CCHMC: Cincinnati Children’s Hospital Medical Center; BCM: Baylor College of Medicine; Boston Children’s Hospital Division of Immunology; Emory University School of Medicine; Duke-NUS Medical School; SickKids: The Hospital for Sick Children; UMDNJ RWJMS: Rutgers Robert Wood Johnson Medical School

**Keywords:** mentorship, workforce, educational quality improvement

## Abstract

**Background:**

The American College of Rheumatology (ACR)/Childhood Arthritis and Rheumatology Research Alliance (CARRA) Mentoring Interest Group (AMIGO) is an inter-institutional mentorship program launched to target mentorship gaps within pediatric rheumatology. Initial program evaluation indicated increased mentorship access. Given the small size of the pediatric rheumatology workforce, maintaining a consistent supply of mentors was a potential threat to the longevity of the network. Our aims were to: (i) describe the sustainability of AMIGO over the period 2011–2018, (ii) highlight ongoing benefits to participants, and (iii) describe challenges in the maintenance of a mentorship network.

**Methods:**

A mixed-methods approach centered on a quality improvement framework was used to report on process and outcomes measures associated with AMIGO annual cycles.

**Results:**

US and Canada Pediatric rheumatology workforce surveys identified 504 possible participants during the time period. As of fall 2018, 331 unique individuals had participated in AMIGO as a mentee, mentor or both for a program response rate of 66% (331/504). Survey of mentees indicated high satisfaction with impact on general career development, research/scholarship and work-life balance. Mentors indicated increased sense of connection to the community and satisfaction with helping mentees despite minimal perceived benefit to their academic portfolios. Based on AMIGO’s success, a counterpart program, Creating Adult Rheumatology Mentorship in Academia (CARMA), was launched in 2018.

**Conclusions:**

Despite the challenges of a limited workforce, AMIGO continues to provide consistent access to mentorship opportunities for the pediatric rheumatology community. This experience can inform approaches to mentorship gaps in other academic subspecialties.

## Introduction

The American College of Rheumatology (ACR)/Childhood Arthritis and Rheumatology Research Alliance (CARRA) Mentoring Interest Group (AMIGO) is a novel subspecialty-wide inter-institutional mentorship program developed to target mentorship gaps within pediatric rheumatology.^1^ This network pairs fellows and junior faculty with more senior faculty mentors at outside institutions based on career interests and goals.^2,3^ Surveys after the initial program implementation revealed increased access to mentorship beyond the home institution and perceived benefits including impact on career development, scholarship, work-life balance, and connectedness to the pediatric rheumatology community for mentees.^3^ Maintaining and growing the mentorship pipeline was the next challenge for the network.

Mentorship during fellowship training and junior faculty years is critical to the development of a successful academic career.^4^ In pediatric rheumatology, the majority of graduating fellows enter into academic positions.^5^ With only approximately 400 practicing board-certified pediatric rheumatologists in the United States and Canada, most programs contain relatively few faculty and the current supply is insufficient to meet clinical demands. Given this workforce shortage, faculty time is often constrained by heavy clinical, research and administrative duties. Local access to senior mentors for fellowship trainees and junior faculty is often limited.^2,5^

The problem of limited access to mentorship is not unique to pediatric rheumatology. In particular, the literature indicates local mentorship resources may be limited for women in certain subspecialties^6,7^ and under-represented minorities^8–10^. Throughout academic medicine there has been a call to develop more formal mentorship programs due to increased awareness of the value of extended mentorship networks to provide diverse insight and expertise.^11–13^

### Educational Challenge:

Based on the success of an initial pilot, AMIGO was formally sponsored by the ACR in 2011. Given the relatively small size of the pediatric rheumatology workforce, maintaining a consistent supply of mentors was foreseen as a potential threat to the longevity of the network. In particular, there exists a ‘bimodal’ age distribution in the pediatric rheumatology workforce with larger numbers of practitioners either at the ending or beginning of their careers and a relatively smaller ‘mid-career’ population; 32% of current Pediatric or Med/Peds Rheumatologists are expected to retire within 10 years.^14,15^ Despite these ‘supply’ challenges, the AMIGO network thrived over these 7 years.

Our specific aims are to: (i) describe the sustainability of the AMIGO mentoring network between 2011–2018, (ii) highlight ongoing benefits to mentees and mentors, and (iii) describe limitations and challenges in the maintenance of a mentorship network. This information will be informative for other subspecialties interested in development of longitudinal mentorship programs.

## Methods

A mixed methods approach was used, drawing on both educational evaluation as well as quality improvement theory.^16,17^ Specifically, the SQUIRE-EDU guidelines was used as a framework.^18^ We used the Kirkpatrick Evaluation Model to report on participant reactions, behaviors and benefit to the organization as a whole ^16^ These responses are situated as process and outcome measures associated with AMIGO annual cycles.

### Context: Program Description

Development and implementation of the AMIGO program has already been reported.^2^ Subsequent to the pilot program, network leadership has been provided by two rotating pediatric rheumatology faculty Co-Chairs. One chair is nominated from the ACR Pediatric Rheumatology Subspecialty Committee, and one is the elected CARRA Early Investigator Committee Vice Chair. The Co-chairs serve three-year terms and are responsible for oversight of the annual matching process, exit surveys, and design, implementation and evaluation of faculty development and networking sessions held during bi-annual scientific rheumatology meetings. ACR staff provides administrative assistance with emails, administration and tabulation of evaluations and surveys and event planning. The ACR also stores survey and evaluation data and maintains the AMGIO website which houses mentorship and professional development resources (https://www.rheumatology.org/Get-Involved/Mentoring/AMIGO). A Microsoft Excel algorithm was used to generate mentee-mentor pairings based on mentee and mentor responses to an annual electronic survey. Demographic information, mentee preferences for career path, research interests, experience and level of mentor seniority were used to generate a list of top matches per mentee. Subsequently, a larger group of volunteer AMIGO committee members conducedt a phone conference to review and adjust algorithmically generated results and ensure the best dyad fit. Dyads were officially followed by the AMIGO program for a three-year period. Participants were sent an anonymous exit survey at the end of the three-year term to obtain more detailed information on utilization of and satisfaction with the program.

### Interventions

AMIGO Co-Chairs and evaluation committee members reviewed data from networking events, email check-ins and the overall program exit evaluations at least semi-annually. Results were used to adjust content and format of educational programing events and to monitor for successful or problematic matches. Starting in 2016 networking events were structured to increase interaction between as well as within dyads attending events to promote extended mentorship networks and informal peer mentorship opportunities.

### Measures and Analysis:

ACR database and surveys were used to obtain data. End of program exit surveys were available from years 2016–2018 reflecting the experience of mentors and mentees who had entered the program in 2012 to 2015 (Kirkpatrick level 1- Reactions). Certain mentors had more than one mentee over the time period and were asked to fill out a separate response for each mentee with whom they were matched.

In terms of **process measures**, we report the number of annual mentee and mentor applications, total active program participants, number of substantive meetings between dyads, and attendance at biannual in-person networking events (Kirkpatrick level 3-Behaviors). **Outcome measures** include: perceived benefit to mentee in specific domains (research/scholarship, teaching, clinical work, career development, negotiating current position or salary, work-life balance), mentee and mentor ratings of dyad ‘goodness of fit’, satisfaction with biannual networking meetings (Kirkpatrick Level 1-Reactions) and number of mentees who return to the network as mentors in subsequent cycles (Kirkpatrick level 3- Behavior). With our interest in mentor sustainability, we also collected additional outcome and **potential balancing measures** (reported impact on mentor’s promotion portfolio, perceived ability to help mentee, and sense of connectedness to the larger pediatric rheumatology community [Kirkpatrick Level 1-Reactions]). To measure impact on the community as a whole (Kirkpatrick level 4- Results/impact) we report on cumulative number of participants in the network relative to the number of fellows and board certified pediatric rheumatologists in the US and Canada, the community served by the program.

Descriptive statistics are used; given the small sample sizes and variable survey response rates, rigorous statistical comparisons were not performed. We were unable to correlate dyad responses or to determine exact denominators for the mentor cohort due to the anonymity of the exit survey.

### Ethical Considerations:

The project was granted exempt status by the Institutional Review Board at Seattle Children’s Hospital (STUDY00000772) as non-human subjects research.

## Results

### Process Measures:

#### Mentee-Mentor dyads:

**Figure 1:**
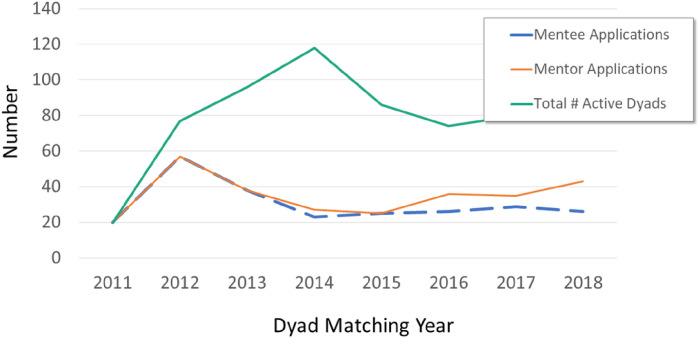
Number of AMIGO mentorship network partcipants over time Growth of AMIGO mentorship network over time

Fig. 1 demonstrates the growth of the network over time. In 2012, the AMIGO program was launched at scale after the initial limited pilot program and thus a large number of mentees joined the program. Subsequently, the annual number of mentee applicants has reached a steady state (median 26; range 20–57) closely paralleling the number of entering US pediatric rheumatology fellows per year (median: 28; range: 23–39) over the time period.^15^ After the initial pilot years, the majority of mentees applicants were fellows, however, some junior faculty members continued to apply to the network (Table 1).

Mentor application numbers remained steady and in certain years exceeded the number of mentee applications.

#### Mentee-Mentor contact:

Exit Survey response rates were variable (for Mentees: 49% [28/57] in 2016; 24% [9/38] in 2017 and 39% [9/23] in 2018; for Mentors: 109% [62/57] in 2016; 53% [20/38] in 2017 and 104% [24/23] in 2018. The greater than 100% response rate observed in mentors was attributed to certain mentors responding separately for multiple mentees who had different dyad starting years.) However, responses indicate that utilization of the AMIGO mentorship network was high. The cohort surveys indicate that dyads connected by phone, email and in-person. Ninety-three percent (43/46) of mentee respondents reported meeting with their mentor at least 2–3 times since the initial match and 72% (33/46) indicated greater than 3 substantive meetings.

### Biannual conferences

Attendance at in person networking events held at ACR and CARRA scientific conferences was high and sustained over time. Table 2 indicates number of respondents to session evaluations which underestimates total attendance at these sessions as not all participants submitted evaluations.

### Outcome measures:

#### Career development sessions

Table 2 also provides highlights from participant evaluations of the biannual networking sessions. Detailed evaluation questions varied between sessions over time. Responses indicating aggregate participant reactions to that day’s overall program or the AMIGO network are included and indicate high ratings for participant satisfaction.

#### Mentee Perceptions

Mentees cited ‘General career development’ as the professional development domain in which AMIGO either ‘helped very much’ or ‘helped somewhat’ (89% [41/46]). The AMIGO relationship was also rated as beneficial in the domains of ‘Research/Scholarship’ (70% [32/46]) and ‘Work-Life Balance’ for most respondents (59% [27/46]). The impact in other specific domains such as ‘Obtaining your current position’ and ‘Negotiating a salary’ was more variable; certain respondents reported the network helped very much (13% [6/46] and 9% [4/46]) while the majority indicated the AMIGO dyad made no difference or these domains were non-applicable to their experience (50% [23/46], 74% [34/46], respectively). Approximately a third of respondents indicated AMIGO ‘helped very much’ or ‘helped somewhat’ for the areas of Clinical work (37% [17/46]) and Teaching (35% [16/46]).

#### Mentee-Mentor fit

**Figure 2:**
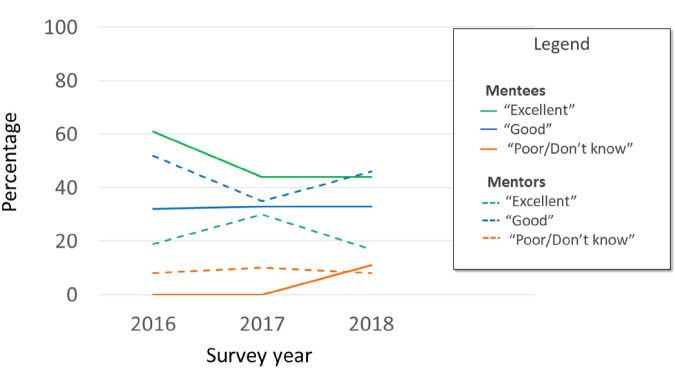
Average rating of ‘goodness of fit’ of mentorship dyad over time by participant status

Results for mentee and mentor perception of ‘goodness of dyad fit’ from cohort surveys are presented in Fig. 2. In general, mentees tended to rate the goodness of fit of the dyad as slightly higher than mentors (most responses “Excellent” or “Good”) however, overtly problematic matches were infrequent over the 3 years. Due to small numbers/variable response rate there was no attempt to perform rigorous comparisons between groups or across years.

#### Mentee to Mentor Transition

As of fall 2018, 25% (40/163) of former mentee participants had returned to the network as mentors in a subsequent cycle.

#### Mentor Perceptions/Balancing measures

**Figure 3:**
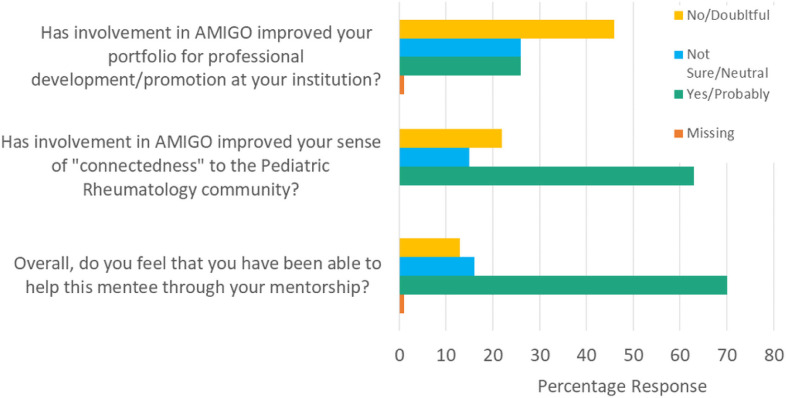
What’s in it for mentors? Aggregate mentor responses to benefit/balancing measures 2016–2018 (n=106)

Figure 3 indicates aggregate mentor responses for all years 2016–2018 (n = 106) to exit survey questions regarding benefits or opportunity costs associated with participation in the network. Most respondents had positive perceptions of their ability to help their mentees and an improved sense of connection to the community, however, the majority also indicated neutral to doubtful benefit for their own academic portfolios.

### Results/Impact on the community

As of fall 2018, 331 unique individuals had participated in AMIGO as mentees, mentors or both. The 2017–2018 American Board of Pediatrics Workforce Survey identified 357 pediatric rheumatologists with active board certification and 96 pediatric or medicine-pediatrics rheumatology fellows.^15^ A 2015 Canadian workforce survey indicates approximately 51 pediatric rheumatologists.^19^ Thus, the total number of possible participants during this time is estimated at 504 individuals. Participation in AMIGO has reached almost 66% (331/504) of the US/Canadian pediatric rheumatology community.

## Discussion

This paper updates our initial reports of an inter-institutional career mentorship program for pediatric rheumatologists.^2,3^ Similar models have now been reported within the pediatric hematology/oncology and trauma surgery communities.^20–22^ Several program evaluations^12,23,24^ and systematic reviews of mentorship models in academic medicine have also been published.^4,25,26^ However, this paper provides the first longitudinal evaluation of an inter-institutional mentorship program over an extended period. We also are unique in the application of an educational quality improvement framework for our evaluation in parallel with classic Kirkpatrick levels of evaluation.

Our findings demonstrate that the AMIGO network was able to sustain stable entry rates and high levels of utilization of and satisfaction. Mentee exit surveys indicate highest levels of satisfaction with impact on domains of General Career Development, Research/Scholarship and Work-Life Balance. In general, both mentees and mentors indicated good-to-excellent ‘fit’ for their match over time. Attendance at and satisfaction with semi-annual networking meetings has been robust. The networking sessions have cultivated an informal environment where participants are able to meet face to face within their assigned dyads as well as to interact with other attendees. In particular, the focus of sessions in 2016–2018 was to promote connections across mentorship dyads in order to foster informal peer mentorship relationships and to grow networks of mentees and mentors with similar interests.

One of the most critical factors important to the long-term success of any mentorship program is sustained access to engaged mentors. In spite of potential supply issues in the senior pediatric rheumatology workforce, the AMIGO network has consistently provided access to all interested mentees, with the number of annual mentor applications exceeding the mentee applications in some years. In part, this is due to certain committed senior mentors who agree to mentor multiple mentees or who consistently reapply after the end of a 3-year cycle. Importantly however, we found that 40 mentee graduates of the program returned to the program as mentors to incoming mentees in subsequent years. This ‘mentee turned mentor’ phenomenon is perhaps one of the key indicators of participant satisfaction with the network and has been critical to sustaining ongoing mentorship access in AMIGO.

In terms of using this experience to replicate mentorship networks in other specialty situations, it will be important to pay attention to cultivation of mentees as future mentors as well as factors that will encourage the recognition and retention of faculty mentors. Interestingly although mentors reported high satisfaction with their participation in the network, there was little perception of benefit towards their academic promotions. Given the competing demands on mid-career faculty, identifying additional ways to increase the tangible impact on mentors’ academic careers will be an important consideration for long term success of these networks. Fortunately, earlier studies within AMIGO had found that the time commitment required for mentors was small, typically a less than 3 hours per year, helping to explain why mentors as well as mentees retain strongly overall satisfaction with AMIGO.^3^

One of the major limitations of our study was the exit survey response rates. This could be attributed to several factors including survey fatigue in our community as well as change in email contact information, particularly for fellow participants who were likely to have graduated and changed institutions over the three-year time-period. Respondents unhappy with or less invested in the program may have been less likely to respond to the surveys. We have retained the anonymity of the exit survey process in an attempt to obtain honest, unbiased feedback on the program, however, this feature also limits our ability to identify non-respondents or to conduct more detailed analyses regarding factors associated with the dyad matching process or the program.

Another potential limitation to the generalizability of our approach is the matching process itself. The initial mentorship dyad matches are created with the use of a Microsoft Excel spreadsheet algorithm, curated through subsequent committee review to ensure face validity of the pairings and revise matches in case of any algorithm failures. This feature is relatively feasible within the pediatric rheumatology community given the small size and close-knit nature of our specialty. Reproducibility within a different or larger community might be more difficult. Lastly, but importantly, this study took place during the pre-pandemic era. Although the AMIGO network continued during the COVID-19 pandemic, both the ACR and CARRA annual meetings were converted to remote formats, which limited opportunities regular for face-to-face interactions are required restructing of the netowork. Additional study will be important to determine how the network and inter-institutional mentorship relationships have been affected by these changes and the increased reliance on virtual connections in the post-pandemic setting.

ACR sponsorship has been a key facet to the sustainability of the AMIGO network. Administrative support in particular has been crucial to the ongoing operation of the network, which would otherwise be solely dependent upon volunteer time from physician leadership. A key indicator of the success of the network is the launch of a parallel network, Creating Adult Rheumatology Mentorship in Academia (CARMA), by the ACR in 2018 to address the burgeoning need for inter-institutional mentorship in adult academic rheumatology.^27^ A parallel program targeted to postdoctoral fellows has been developed within the NIH-funded Joint Biology Consortium research infrastructure https://www.jbcwebportal.org/jbc-mentorship-program/. The ACR is also developing a structured mentorship program for division directors within the organization (M. Klein-Gitelman, personal communication, 7/21/2020). Replication in other settings would likely require identification of a similar funding or sponsorship agency.

## Conclusion

Over the span of seven years, AMIGO has provided much-needed access to inter-institutional mentorship for pediatric rheumatologists across the United States and Canada. More than 66% of the Pediatric Rheumatology community has been involved in this program with a high proportion of program graduates returning to the network in a mentorship role. Future ideas for improvement include optimizing exit survey response rates, expanding access to diversity mentorship and exploring the impact of peer mentorship in addition to the more formal vertical mentorship structure. We hope our process and learning can facilitate the development of other programs to improve access to mentorship within underserved pediatric communities.

## Figures and Tables

**Table 1:** Demographics for AMIGO annual mentor-mentorship dyad matching process over time

Match/Entry Year	2011	2012	2013	2014	2015	2016	2017	2018
**Mentee Applications (N)**	20	57	38	23	25	26	29	26
Gender								
Female (%)	70	82.5	76	83	80	77	79	77
Nationality								
Canadian (%)	0	9	8	4	4	4	3	12
Academic Rank								
Fellows (%)	85	82	97	100	84	92	93	92
Jr. faculty* (%)	15	18	3	0	16	4	7	4
Sr. faculty**(%)	0	0	0	0	0	0	0	0
Unknown (%)	0	0	0	0	0	4	0	4
**Mentor Applications (N)**	20	57	38	27	25	36	35	43
Gender								
Female (%)	70	58	58	59	60	61	71	63
Nationality								
Canadian (%)	10	8.8	5.3	0	4	2.8	8.6	0
Academic Rank								
Jr. faculty* (%)	15	16	45	52	40	58	60	56
Sr. faculty**(%)	60	30	47	48	60	39	40	40
Unknown (%)	30	54	8	0	0	3	0	5

**Table 2:** AMIGO biannual faculty development and networking session attendance and participant overall ratings

Meeting: AMIGO Event Title	Evaluation Question(s)	Rating (scale 1-5)*	Respondents (n)**
2014 CARRA Session	• *Potential value of the AMIGO program?*	1.6*	57
2015 CARRA Session: "Promotions Bootcamp"	• *Understanding of promotion domains after session?*	4.3	43
	• *Grasp of career development documentation after session?*	4.1	43
2015 ACR breakfast: "Mentorship Resource Fair"	• *Value of today’s mentorship resource fair?*	4.7	33
	• *Value of encouraging mentorship networks?*	4.8	33
2016 CARRA session: "Mentorship 360°"	• *Talk: "Mentoring 360° "*	4.7	43
	• *Potential value of a mentoring program as envisaged by AMIGO?*	4.8	43
2016 ACR breakfast: "Building Your Academic Career"	• *Value of today's mentorship talk?*	4.7	12+
	• *Value of encouraging mentorship networks?*	4.8	12+
2017 CARRA/PRYSM: "Peer mentorship"	• *Value of AMIGO's current vertical mentorship program?*	4.7	36
	• *Value of a potential Peer/Horizontal mentorship program?*	4.3	36
2017 ACR Breakfast: "'GROW#' your mentorship network"	• *Value of today’s mentorship resource fair?*	4.8	37
	• *Value of encouraging mentorship networks?*	4.8	37
2018 CARRA Breakfast	• *Value of today’s networking session ?*	4.8	32
2018 ACR Breakfast	• *Value of today’s networking session ?*	4.8	40
	• *Value of encouraging mentorship networks?*	4.8	40

* 5 = Strongly agree except for 2014 where 1 = strongly agree

** Responses from session attendees, sessions were open to AMIGO participants and non-participants

+ Actual attendance estimated at 35–40 participants (author KH observation) however, evaluations were sent electronically after the event and only 12 responses were received

## Data Availability

The data that support the findings of this study are available from the American College of Rheumatology (ACR) but restrictions apply to the availability of these data, which were used under license for the current study, and so are not publicly available. Data are however available from the authors upon reasonable request and with permission of the ACR.

## Abbreviations

ACR: American College of Rheumatology
CARRA: Childhood Arthritis and Rheumatology Research Alliance (CARRA)
AMIGO: American College of Rheumatology (ACR)/Childhood Arthritis and Rheumatology Research Alliance (CARRA) Mentoring Interest Group
SQUIRE-EDU: Standards for QUality Improvement Reporting Excellence in Education
GROW: Goal Reality Options Will Mentorship Model
